# Impact of COAP on access to primary care in Ceará and Mato Grosso do Sul through the analysis of interrupted time series

**DOI:** 10.11606/s1518-8787.2021055003001

**Published:** 2021-04-23

**Authors:** Débora Castanheira Pires, Mônica Rodrigues Campos, Isabel Martins Emmerrick

**Affiliations:** I Fundação Oswaldo Cruz Instituto de Comunicação e Informação Científica e Tecnológica em Saúde Laboratório de Informação em Saúde Rio de JaneiroRJ Brasil Fundação Oswaldo Cruz. Instituto de Comunicação e Informação Científica e Tecnológica em Saúde. Laboratório de Informação em Saúde. Rio de Janeiro, RJ, Brasil; II Fundação Oswaldo Cruz Escola Nacional de Saúde Pública Sergio Arouca Departamento de Ciências Sociais Rio de JaneiroRJ Brasil Fundação Oswaldo Cruz. Escola Nacional de Saúde Pública Sergio Arouca. Departamento de Ciências Sociais. Rio de Janeiro, RJ, Brasil; III University of Massachusetts Medical School Department of Surgery Division of Thoracic Surgery WorcesterMassachusetts USA University of Massachusetts Medical School. Department of Surgery. Division of Thoracic Surgery. Worcester, Massachusetts, USA

**Keywords:** Unified Health System, Regional Health Planning, Primary Health Care, Evaluation Studies as Topic, Program Evaluation

## Abstract

**OBJECTIVE:**

To analyze the impact of implementing the *Contrato Organizativo de Ação Pública* (COAP – Public Action Organizational Contract) on the expansion of access to primary care in the states of Ceará and Mato Grosso do Sul.

**METHODS:**

We used the interrupted time series method to analyze the effect of COAP on primary care coverage (PCCov) and on avoidable hospitalization rates. To analyze the effects of increased PCCov on avoidable hospitalizations, we used non-segmented time series models.

**RESULTS:**

The results showed that implementing COAP had a positive impact on increased coverage in both cases, with did not happen in the control states. However, this impact was not reflected in the decrease in hospitalizations due to primary care sensitive conditions (HPCSC) or for acute preventable causes. When we analyzed the effects of the increase in PCCov on avoidable hospitalizations between 2009 and 2016, we observed that coverage had a positive impact on the decrease in the rate of HPCSC only in Ceará, although hospitalizations have a significant trend to decrease in time both in this state and in Mato Grosso do Sul, except for acute respiratory infections.

**CONCLUSIONS:**

The COAP continues to be the regulatory instrument of regionalization force, however, the results obtained by adhering to it in the expansion of primary care in Ceará and Mato Grosso do Sul makes us question whether the contractual model, as predicted, is the best instrument for advancing regionalization in the Brazilian Unified Health System.

## INTRODUCTION

The *Contrato Organizativo de Ação Pública* (COAP – Public Action Organizational Contract) was created by Decree 7.508/2011^1^ to regulate Law 8.080/1990 and provide legal support to the process of inter-federative coordination of the Brazilian Unified Health System (SUS), thus facilitating the necessary agreements to maintain the tripartite responsibilities of health administration. Between 2011 and 2016, the Ministry of Health undertook efforts to build the pact around the COAP throughout the country^2^. The contractual model, in which responsibilities and goals are established between Federative entities, was considered ideal to accommodate the organization of the SUS^3^.

However, the COAP was only effective for the states of Ceará and Mato Grosso do Sul. Among the obstacles to complete the contracting process, the difficulty of the state health secretariats in coordinating the process, the complexity of the instrument, the insufficient, dispersed and inadequate funding for implementing regional systems^2^^,^^4^ and the high transaction costs in joining the contract stand out^5^. In the states where it was finalized, however, the impacts on the health area have not been studied to date. This is even more worrying because Decree 7,508/2011 remains in force and, to date, is the regionalization directive in force in the SUS.

This article aims to analyze the impact of the implementation of COAP on the expansion of access to primary care precisely in the states where it was finalized, Ceará and Mato Grosso do Sul.

## METHODS

### Intervention

The COAP is a legal-executive device introduced in the normative framework of the SUS by Decree 7.508/2011^1^ with the aim of regulating the planning and inter-Federative articulation of the health system. Only Ceará and Mato Grosso do Sul have finalized the negotiation process to implement the COAP, which was negotiated with municipalities and regions throughout 2012 and entered into force in 2013.

### Design and Data Source

This is a retrospective, quantitative and analytical study that uses the time series data regression method. The study will have as its source administrative data from the *Sistema de Internações Hospitalares do SUS* (SIH-SUS – Hospitalization System of SUS) and the *Sistema de Cadastro Nacional de Estabelecimentos de Saúde* (SCNES – National Registry System of Health Establishments), as well as population data from the demographic censuses of the Brazilian Institute of Geography and Statistics (IBGE) conducted over the period studied and projections.

### Control Selection Methodology

We chose to construct models using controls recommended for interrupted time series (ITS)^6^ studies, but in order for the results to be reliable, this approach should use structurally similar sites^7^. Thus, in the selection of controls we used a set of indicators, which were divided into five socioeconomic and demographic indicators, and four health systems. The year 2011 was used as a reference for obtaining information, as it was the last year before the implementation of COAP. The only exception was the Human Development Index - Municipal (HDI-M), estimated on the basis of United Nations Development Program Report (UNDP 2010).

Brazilian states were divided into groups according to the degree of implementation of the COAP^4^, and controls were chosen among the group that had bureaucratic implementation, because these states seemed to have technical and political capacity to put the program into practice. The category of bureaucratic implementation is defined by the existence of state responses concentrated in groups of chained moments of the implementation agenda. Thus, it is considered that the state fulfilled the bureaucratic implementation when it carried out the stages of construction of the initial pact, reconfiguration of the health regions and established *Comissões Intergestoras Bipartites Regionais* (CIR – Regional Bipartite Intergovernmental Commissions) or Regional Planning.

To estimate the summary index that guided the selection of controls, we used the difference between the values of the reference states ' Ceará and Mato Grosso do Sul that had the implementation finalized ' and the values of other states (Formula 1). From these values, the mean of the difference of each of the states and the standard deviation were estimated.

### Formula 1

β = *Cs – Rs*

In which: Cs = Control state and Rs: Reference state

Then, for each indicator, the amount of standard deviations that each state was from the mean of the difference was estimated, thus determining the similarity between the possible controls and the respective cases (Formula 2).

### Formula 2

γ = (β – *M*)/*DP*

*In which:* β *= difference data; M = mean; DP = standard deviation*

Finally, the absolute value of γ, of each indicator per state, was added. The states that had the result of the sum closest to zero were the closest to the reference states. Thus, the control for Ceará was the state of Sergipe (γ = 3.26) and for Mato Grosso do Sul the control was the state of Mato Grosso (γ = 2.20).

### Selection of Outcome Indicators

Indicators classified as universal in the contract ' that is, mandatory for all states, health regions and municipalities ' and whose information was available with free access in the *Sistemas de Informação da Saúde* (SIS – Health Information Systems) were used for the impact assessment of the COAP. In order to evaluate the expansion of access to primary care, we selected indicators of primary care coverage (PCCov) and the rate of hospitalizations due to primary care-sensitive conditions (HPCSC), belonging to Guideline 1, National Objective 1.1 of COAP^1^, considering the monthly availability of data and their sensitivity to the actions provided for in the specific objectives.

Considering the rates of HPCSC, provided in the COAP, the hospitalization rate was calculated according to the groups of causes considered “acute”, in order to capture those on which the changes occurred in primary care could have a more immediate effect, such as gastroenteritis, acute respiratory infections (ARI) and asthma^8^.

Looking for a more stable measure in the indicators, we evaluated different periodicities. In PCCov, the monthly periodicity was used, while, for indicators of hospitalization, a monthly periodicity was applied in the case of Ceará and Sergipe, and quarterly for Mato Grosso do Sul and Mato Grosso.

### Statistical Analysis

We used ITS models to analyze the effect of COAP on outcome variables. When estimating effects, these models adjust for pre-existing trends to the intervention. The Prais-Winsten estimate was used in the STATA v. 12 software to perform regression analyses.

The models used two time segments, the period prior to signing (January 2009 to December 2011) and post COAP (January 2013 to December 2016), considering 1 year for the implementation of the program (January to December 2012). The effects of COAP were estimated using one variable representing the change in the level of the outcome variables immediately after its implementation and another representing the change in trend post-intervention.

To analyze the effects of increased PCCov on avoidable hospitalizations over time, we used non-segmented time series models, employing Prais-Winsten regression in STATA v.12.

In both cases, model parameters were preserved regardless of statistical significance. Results with p < 0.05 were highlighted. The models for the existence of autocorrelation of residues were adjusted using the Durbin-Watson test^9^. We also tested logarithmic trend terms to accommodate possible nonlinear trends during the post-intervention segment, selecting the best model using the Bayesian information criteria (BIC) and Akaike information criteria (AIC)^10^.

To create single-number summaries of the effects of the policies, the percentage of relative change, we estimated the relative changes in the results of January 2013, shortly after the implementation of the COAP, compared with the expected values based on trends prior to the implementation of the contract.

This study uses only secondary databases of open access and is therefore exempt from approval by the Research Ethics Committee.

## RESULTS

In 2011, Ceará had a moderate degree of urbanization^11^, average Human Development Index (HDI)^12^, with an aging index and life expectancy at birth lower than the national average for the same year. The control, Sergipe, shared these characteristics, despite having a less aged population. As for the health system, the number of doctors per thousand inhabitants of Ceará was much lower than the Brazilian average, but the number of beds for hospitalization was very close to the national one. Sergipe had more doctors per inhabitant, but remained below the national average, with fewer hospital beds available. Supplemental health coverage in both states was very similar (below the national average). The biggest difference between the two is in PCCov, which in Ceará was a little lower than that of Brazil, while in Sergipe it was quite superior.

Mato Grosso do Sul and its control, Mato Grosso, had a high degree of urbanization, with high HDI and an aging index lower than the national average. Life expectancy at birth in Mato Grosso do Sul was exactly the same as in Brazil, while in Mato Grosso it was slightly lower. The number of physicians per capita was also similar between states, both below the national average, the number of beds per inhabitant and PCCov were very close to national values for both. Supplemental health coverage was lower than Brazilian coverage in both, and higher in Mato Grosso do Sul than in Mato Grosso (Table 1).

**Table 1:** Sociodemographic and health care network indicators for Brazil (BR), Ceará (CE), Sergipe (SE), Mato Grosso do Sul (MS) and Mato Grosso (MT) in 2011.

		CE	MS	SE	MT	BR
SOCIODEMOGRAPHIC	Population ageing index	35.3	35.5	29.0	28.8	41.2
	Sex ratio	95.1	99.3	94.5	104.3	96.0
	Life expectancy at birth	72.7	74.1	71.3	72.9	74.1
	HDI^a^	0.68	0.73	0.67	0.73	0.7
	Urbanization degree	75.5	85.8	73.6	82.0	84.4
HEALTH	Number of doctors per thousand inhabitants^2^	0.9	1.4	1.2	1.1	1.9
	Primary care coverage	66.7	70.1	83.4	67.9	67.1
	Number of hospital beds per thousand inhabitants^b^	2.2	2.3	1.9	2.2	2.4
	Supplementary health coverage	12.2	17.2	13.3	12.9	23.5

a Values based on UNDP 2010.

b Values from December 2011.

Source: Demographic census and projections of IBGE, UNDP (2010), *Sistema de Cadastro Nacional de Estabelecimentos de Saúde* (SCNES) and *Sistema de Informação de Beneficiários* (SIB/ANS/MS).

As can be seen in Table 2, PCCov in the period prior to the implementation of COAP (January 2009 to December 2011) had a slight downward trend in Ceará and Sergipe and also a slight upward trend in Mato Grosso do Sul and Mato Grosso. After the implementation of COAP, Ceará and Mato Grosso do Sul showed significant increases in the indicator.

**Table 2:** Models of analysis of the effectiveness of COAP on PCCov and on the rates of avoidable hospitalizations through STIs.

Indicator	FU	Baseline			Post COAP (Jan. 2013)		Value on Jan. 2013	Relative change (Jan. 2013)
		Value on Jan. 2009	Trend	Value on Dec. 2011	Post COAP level (95%CI)	Post COAP trend (95%CI)		
	**CE**	78.75	-0.10	75.33	**8.97 (2.57 to 15.37)**	0.13 (-0.10 to 0.36)	83.16	**4.52**
Primary care coverage^b^	SE^a^	91.70	-0.09	88.47	1.52 (-2.91 to 5.95)	0.09 (-0.04 to 0.23)	88.89	1.88
	**MS**	65.45	0.01	65.74	**5.26 (1.19 to 9.33)**	0.02 (-0.12 to 0.16)	71.13	**8.63**
	**MT**^a^	68.54	0.06	70.67	**-3.02 (-5.15 to −0.89)**	0.05 (-0.02 to 0.12)	68.50	-6.66
	CE	8.74	-0.03	7.59	0.40 (-1.03 to 1.83)	0.00 (-0.04 to 0.05)	7.57	1.88
HPCSC rate^c^	SE^a^	4.30	-0.02	3.65	0.23 (-0.6 to 1.05)	0.02 (-0.01 to 0.05)	3.65	3.33
	MS	27.43	-0.17	25.54	-0.09 (-4.93 to 4.76)	0.12 (-0.36 to 0.59)	24.72	0.68
	MT^a^	28.77	-0.27	25.78	-1.84 (-4.31 to 0.64)	0.10 (-0.15 to 0.35)	22.69	-1.86
	CE	1.60	-0.02	0.95	0.66 (-0.33 to 1.64)	0.01 (-0.02 to 0.05)	1.37	3.36
Avoidable hospitalization rate by ARI^c^	**SE**^a^	0.34	0.01	0.69	-0.16 (-0.52 to 0.2)	-0.01 (-0.02 to 0)	0.68	**5.79**
	MS	3.99	-0.06	3.31	2.36 (-0.01 to 4.74)	0.03 (-0.22 to 0.28)	5.39	2.07
	**MT**^a^	5.63	-0.31	2.18	**2.45 (0.71 to 4.18)**	**0.31 (0.13 to 0.49)**	3.38	**5.89**
	CE	1.31	-0.01	0.85	0.10 (-0.22 to 0.41)	0.00 (-0.01 to 0.01)	0.78	-3.69
Avoidable hospitalization rate by asthma^c^	SE^a^	0.48	0.00	0.40	0.09 (-0.18 to 0.36)	0.00 (-0.01 to 0.01)	0.46	5.47
	**MS**	3.88	-0.23	1.39	0.78 (-0.72 to 2.29)	**0.19 (0.03 to 0.35)**	1.22	-2.72
	**MT**^a^	3.52	-0.13	2.04	-0.26 (-1.04 to 0.52)	**0.10 (0.02 to 0.18)**	1.21	**2.01**
	CE	2.92	-0.03	1.89	0.69 (-0.55 to 1.93)	0.01 (-0.03 to 0.05)	2.20	4.15
Avoidable hospitalization rate by gastroenteritis^c^	**SE**^a^	1.19	-0.02	0.57	**0.31 (0.03 to 0.6)**	**0.01 (0 to 0.02)**	0.67	**4.29**
	MS	8.45	-0.17	6.59	-0.59 (-3.52 to 2.34)	0.13 (-0.16 to 0.42)	5.29	-0.19
	MT^a^	7.63	-0.04	7.14	-2.23 (-4.97 to 0.51)	-0.04 (-0.31 to 0.23)	4.65	-15.36

HPCSC: hospitalizations due to primary care-sensitive conditions; ARI: Acute respiratory infections.

a Control cases.

b ((no. of ESF + no. of equivalent ESF) x 3,000/population in the same place and period) x 100. The estimation of the population covered by primary care has as a reference three thousand people per primary care team, according to the Política Nacional de Atenção Básica (PNAB – National Policy of Primary Care), (Ordinance No. 2,488/11).

c Hospitalization models for MS and MT were estimated with quarterly data as a function of the monthly variation.

Source: *Sistema de Internações Hospitalares* (SIH), *Sistema de Cadastro Nacional de Estabelecimentos de Saúde* (SCNES) and demographic census and projections of IBGE.

Soon after the intervention, PCCov in Ceará had a significant increase of 9 percentage points, which did not happen in Sergipe. It is also possible to observe that the result achieved in Ceará in January 2013 is significantly higher (about 4.5%) than that estimated in case of continuity of the trend prior to the implementation of COAP (going from 79% to 83%). In Sergipe, as expected, there is no significant change between the estimated scenario (counterfact) and the real one.

The PCCov level in Mato Grosso do Sul had a significant increase of 5.3 percentage points immediately after the intervention. In Mato Grosso we see a significant drop of 3.0 percentage points between December 2011 and January 2013. The model also shows that coverage in Mato Grosso do Sul in January 2013 was 8.6% higher than what would be achieved in case of maintaining the pre-implementation scenario (going from 65% to 71%).

The effectiveness of primary health care is often evaluated from indicators of avoidable hospitalization^13^, and in this line, COAP has provided its own indicator for this purpose. In the pre-intervention period, it was possible to observe a downward trend in the rate of HPCSC for all states, which was changed to an increase trend, not significant, after the intervention in all states.

The rate of avoidable hospitalization for ARI in the period prior to the intervention had a slight trend of decrease in Ceará and Mato Grosso do Sul, and a slight trend of increase in Sergipe. Mato Grosso had a more marked downward trend in the period prior to 2011. After the intervention we did not find significant changes in Ceará or Sergipe, however, in this we see a relative change percentage of 5.8% in relation to the counterfact. This can be explained because the estimation of the relative change has as variable the value of the indicator at the beginning of the time series, and it doubles in the period from 2009 to 2011 (prior to implementation), which in the general estimation is considered as a significant change to the predicted values, but this is not associated with the intervention.

No significant changes were observed after the intervention in Mato Grosso do Sul. In Mato Grosso we see significant increase in level and trend in the period after the first quarter of 2013, with a relative change percentage of 5.9%.

The rate of avoidable hospitalization for asthma had a downward trend in Ceará, Mato Grosso do Sul and Mato Grosso. After the intervention, the model shows a trend of non-significant increase in Ceará and Sergipe. In Mato Grosso do Sul, we see a significant increase trend in the post-intervention period, but this does not reflect in significant relative change. In Mato Grosso, it is also possible to observe a significant increase trend, with a relative change percentage of 2.0% (p < 0.05).

Regarding the avoidable hospitalization rate for gastroenteritis, all states showed a downward trend between January 2009 and December 2011. After implementation, we see no changes in Ceará, but in Sergipe the period shows significant increase in level and trend, with a relative change percentage of 4.3%. The model did not find significant changes for Mato Grosso do Sul and Mato Grosso.

**Figure 1:**
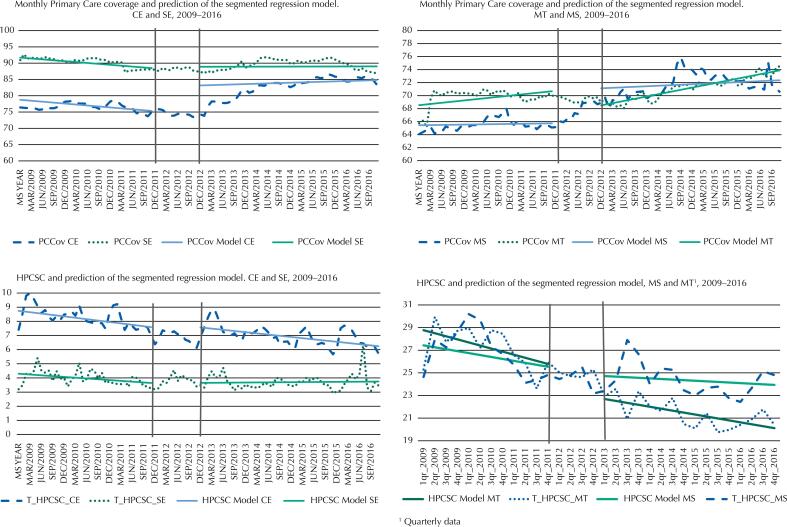
Primary care coverage, HPCSC rate and predicted values of the segmented regression model.

Sources: SIH-SUS and demographic census and projections, IBGE.

Figure 1 shows the visual representation of the impact models of COAP on PCCov and the rate of HPCSC, for cases and controls. It is possible to clearly observe the increase in PCCov level in Ceará, to the detriment of the stability of the indicator observed in the control. In the same way, the coverage in Mato Grosso do Sul increases noticeably in level, while in Mato Grosso we observe a sharp drop. It is important to note that there is a significant difference between the behavior of case trends and post-intervention control, with stability for the control and growth of cases.

For the rate of hospitalization by HPCSC, we see that the pre-and post-COAP scenarios remain practically unchanged in Ceará and Sergipe. It is possible to see a small increase in the level of hospitalizations for Mato Grosso do Sul and a decrease in the sharp downward trend present before the implementation of the COAP, which in the statistical model appeared as a non-significant upward trend (Figure 1).

Therefore, COAP impacts on PCCov were found in the cases, but not an expected drop in HPCSC rates. Considering that literature indicates that the increase in PCCov is correlated with the decrease in hospitalizations due to this set of causes^14^^,^^15^, simple time series models were developed to analyze the impact of the expansion of coverage on hospitalizations over time.

Table 3 summarizes the results of the analysis models of PCCov's influence over time on avoidable hospitalizations for Ceará and Mato Grosso do Sul. It was shown that, although avoidable hospitalizations in Ceará declined significantly over time in almost all sets of causes (with the exception of avoidable hospitalizations due to ARI), the increase in PCCov was significant only in the decrease in the rate of HPCSC.

**Table 3:** Models of analysis of the influence of primary care coverage and time on avoidable hospitalizations in Ceará (CE) and Mato Grosso do Sul (MS), 2009–2016

Indicator	Coverage					Time			
	FU	Coef.	p > t	95%CI		Coef.	p > t	95%CI	
HPCSC rate	**CE**	**9.27**	**0.04**	**0.51**	**18.03**	**-0.03**	**0.00**	**-0.05**	**-0.02**
	**MS**^a^	0.08	0.75	-0.43	0.59	**-0.19**	**0.05**	**-0.37**	**0.00**
Avoidable hospitalization rate by ARI	CE	1.33	0.55	-3.09	5.75	0.00	0.40	-0.01	0.00
	MS^a^	0.15	0.37	-0.18	0.48	0.03	0.60	-0.08	0.14
Avoidable hospitalization rate by asthma	**CE**	0.67	0.49	-0.69	3.07	**-0.01**	**0.00**	**-0.01**	**-0.01**
	**MS**^a^	0.12	0.22	-0.07	0.31	**-0.12**	**0.01**	**-0.16**	**-0.03**
Avoidable hospitalization rate by gastroenteritis	**CE**	6.18	0.12	-0.97	15.09	**-0.02**	**0.00**	**-0.04**	**-0.01**
	MS^a^	-0.08	0.63	-0.44	0.27	-0.10	0.11	-0.23	0.02

HPCSC: hospitalizations due to primary care-sensitive conditions; ARI: acute respiratory infections.

a Hospitalization models for MS were estimated with quarterly data as a function of the monthly variation.

Source: *Sistema de Internações Hospitalares* (SIH), *Sistema de Cadastro Nacional de Estabelecimentos de Saúde* (SCNES) and demographic census and projections of IBGE.

In Mato Grosso do Sul the results were similar. While hospitalization rate drops significantly over time for HPCSC and asthma, the increase in coverage was not significant in any of the cases (Table 3).

**Figure 2:**
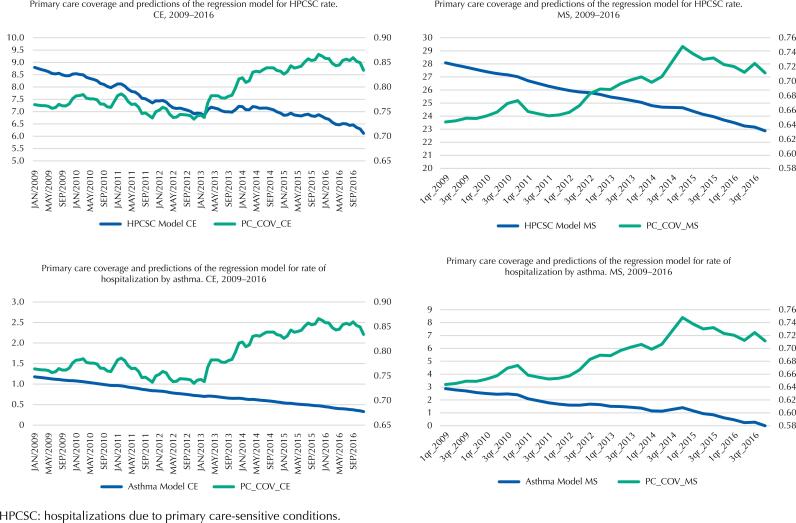
Primary care coverage and results of regression models for avoidable hospitalizations (total and asthma) in Ceará (CE) and Mato Grosso do Sul (MS), 2009–2016.

Source: SIH-SUS, SCNES-SUS and demographic census and projections

These results may be best observed in Figure 2, which shows a jump in PCCov in 2013, especially visible in the state of Ceará, and which is not accompanied by an equally significant drop in hospitalization rates. Thus, despite the increase in PCCov in the state in the post-intervention period, the downward trend in the rate of HPCSC slows down in the same period, only falling again more sharply from March 2015. The rate of avoidable hospitalization for asthma in Ceará does not seem to have been altered. In Mato Grosso do Sul, it is also possible to observe an increase in PCCov level that is not accompanied by any change in the trend of decrease in the rate of HPCSC or asthma.

## DISCUSSION

This article uses ITS to verify the impact of COAP in Ceará and Mato Grosso do Sul. The analysis used control cases for the two states, since the use of controls is important when using ITS due to the difficulty of inferring causality between the data patterns found and the intervention studied^6^.

The results showed that the implementation of COAP had a positive impact on the increase in coverage in both cases, which did not happen in the controls. However, this impact was not reflected in the decrease in the rates of HPCSC or hospitalizations for acute preventable causes. When we analyzed the effects of the increase in PCCov on avoidable hospitalizations between 2009 and 2016, we observed that coverage positively impacted the decrease in the rate of HPCSC only in Ceará, although hospitalizations have a significant trend of decrease in time both in this state and in Mato Grosso do Sul, except for acute respiratory infections.

The models show that immediately after the implementation of COAP, the level of PCCov increases significantly in Ceará and Mato Grosso do Sul. Since the increase occurs in January 2013 and does not occur in the controls, we can rule out that these results are a confusion with possible impacts of competing programs, especially the Programa Mais Médicos, started in July 2013^16^.

Coverage is the most sensitive indicator to assess the increase in access to basic health services, since it precisely measures the availability of these services in the territory. This data is important because it indicates an expansion of access to health services, since primary care is, in Brazil, the main strategy for coordinating care in the network^17^.

The rate of hospitalization by HPCSC, in turn, is widely used to measure the performance of primary care, considering that high rates of hospitalization for certain diseases reflect problems and difficulties in accessing health services and low resolution of primary care^13^.

Due to the type of intervention, it would be expected that there would be no change in the level of hospitalizations by HPCSC soon after implementation, since the increase in PCCov and the decrease in rates do not occur simultaneously^18^. What we could expect was an increase in the downward trend after the implementation of COAP, but this was not observed.

The models showed no significant impact of the COAP on the decrease in the rates of HPCSC or on the rates of hospitalization for acute preventable causes. However, considering the results of the models that indicate that COAP had an impact on the increase in PCCov in cases, analyses were prepared to estimate the specific effects of the increase in PCCov on avoidable hospitalizations between 2009 and 2016.

The rate of HPCSC in Ceará falls significantly both in time and in relation to the increase in PCCov. This corroborates other studies on the state, which correlated the increase in the coverage of the Estratégia de Saúde da Família to the decrease in the rate of hospitalizations due to primary care-sensitive conditions (HPCSC)^19^. The HPCSC rate in Mato Grosso do Sul falls significantly over time but is not correlated with the expansion of PCCov. This indicates that there are other determinants of hospitalization, which are outside the scope of primary health care, such as characteristics inherent to the patient, socioeconomic and demographic factors, variability of hospital clinical practice, and admission policies in these services^20^^,^^21^, in addition to the health care organization model^19^^,^^22^. In this sense, the HPCSC rate is a less sensitive and specific indicator to the analyzed phenomenon^23^.

Avoidable hospitalization rates for ARI were not significantly associated with time and increase in PCCov in both states. On the other hand, hospitalizations for asthma fall significantly in time in both cases. This may be related to the distribution of asthma drugs free of charge by the popular pharmacy program from June 2012^24^.

The application of the ITS method, an important tool in measuring the effect of public policies, stands out as a novelty of this study^25^^,^^26^. Most of the articles on the topic are aimed at understanding the reasons for low adherence to COAP^2^^,^^4^^,^^5^^,^^27^. However, in the perception of the state administrators of Ceará, the results achieved fell short of those expected^28^.

Sergipe was chosen as control due to its greater mathematical similarity, considering the selected indicators. However, as a limitation, we highlight the possibility of a “ceiling effect” for the PCCov indicator and possible “floor effect” in hospitalizations for ARI and asthma.

There is a gap in literature regarding the analysis of the HPCSC or ICSAP for Mato Grosso do Sul in the reference period of this study, despite the discussion being found for all other states of the country.

This article evaluated only the expansion of primary care versus COAP. Therefore, we suggest that similar studies should be conducted to evaluate the impact of the contract on other dimensions. COAP is not the first attempt to create an instrument to mediate regionalization in health (e.g. NOB-SUS 01/91, 01/93 and 01/96; NOAS-SUS 01/01 and 01/02; Pacto pela Saúde), so the use of ITS can be a tool to evaluate previous attempts at regional integration of SUS.

## CONCLUSIONS

The successful decentralization of SUS throughout the 1990s transferred to municipalities a large part of the functions of resource management and implementation of health policies and programs^4^. The deepening of this process led to the fragmentation of the health system and the need to create instruments that guarantee the organization of care networks^5^.

COAP was adopted in 2011, after various attempts made in this direction during the 1990s and 2000s did not achieve the expected results^3^^,^^27^, since, in theory, it would be a proposal more compatible with the Federative model, as it would allow Federated entities in the health region to self-regulate, defining themselves the distribution of executive, budgetary-financial and control and evaluation competences.

Adherence to COAP was, however, very low. The Ministry of Health was not able to create a chain of incentives that had the power to convince and induce others involved in contractualization^2^^,^^5^, as well as to organize internally to govern with cohesion the regionalization process^28^. Despite initiatives to create thematic health networks, such as the QualiSUS-Rede project, these were not related to COAP^29^.

This study showed that COAP had a positive impact on PCCov, an indicator of structure, naturally more sensitive to the direct action of the public administrator. This did not translate into a decrease in the rates of HPCSC, a performance indicator designated in the COAP itself, which is, however, less sensitive and specific to the type of change analyzed.

In December 2016, both Ceará and Mato Grosso do Sul did not renew the COAP, which exhausted the initiative. However, this remains the regulatory instrument of regionalization in force. Considering that the need for regional coordination continues to exist, and that some bills seek to reactivate it^30^^,^^31^, the adequacy of the contractual model to advance the regionalization of SUS should be evaluated from a cost-benefit perspective, assessing, on the one hand, the low adherence to COAP caused by high transaction costs and, on the other, the results obtained by adherence on the expansion of primary care in Ceará and Mato Grosso do Sul.
