# Supplementary material for: Impact of COAP on access to primary care in Ceará and Mato Grosso do Sul through the analysis of interrupted time series
Source: Rev Saude Publica. 2021 Apr 23;55:20. doi: 10.11606/s1518-8787.2021055003001 (PMC8064654; doi:10.11606/s1518-8787.2021055003001)
Supplement: Translation

# Translation: Impacto do COAP sobre o acesso à atenção básica no Ceará e Mato Grosso do Sul por meio da análise de séries temporais interrompidas

**Authors:** Débora Castanheira Pires, Mônica Rodrigues Campos, Isabel Martins Emmerrick

**Affiliations:** I Rio de JaneiroRJ Brasil Fundação Oswaldo Cruz. Instituto de Comunicação e Informação Científica e Tecnológica em Saúde. Laboratório de Informação em Saúde. Rio de Janeiro, RJ, Brasil; II Rio de JaneiroRJ Brasil Fundação Oswaldo Cruz. Escola Nacional de Saúde Pública Sergio Arouca. Departamento de Ciências Sociais. Rio de Janeiro, RJ, Brasil; III WorcesterMassachusetts USA University of Massachusetts Medical School. Department of Surgery. Division of Thoracic Surgery. Worcester, Massachusetts, USA

**Keywords:** Sistema Único de Saúde, Regionalização, Atenção Primária à Saúde, Estudos de Avaliação como Assunto, Avaliação de Programas e Projetos de Saúde

## Abstract

**OBJETIVO:**

Analisar o impacto da implementação do Contrato Organizativo de Ação Pública (COAP) sobre a ampliação do acesso à atenção básica nos estados do Ceará e Mato Grosso do Sul.

**MÉTODOS:**

Utilizou-se o método de séries temporais interrompidas para analisar o efeito do COAP sobre a cobertura da atenção básica (CobAB) e sobre as taxas de internação evitáveis. Para analisar os efeitos do aumento da CobAB sobre as internações evitáveis ao longo do tempo, foram utilizados modelos de séries temporais não segmentadas.

**RESULTADOS:**

Os resultados mostraram que a implementação do COAP teve impacto positivo sobre o aumento da cobertura nos dois casos, o que não aconteceu nos controles. Contudo, esse impacto não se refletiu na diminuição das taxas de internação por condições sensíveis à atenção básica (ICSAB) ou por causas evitáveis agudas. Quando analisamos os efeitos do aumento da CobAB sobre as internações evitáveis entre 2009 e 2016, observamos que a cobertura teve impacto positivo na diminuição da taxa de ICSAB apenas no Ceará, apesar de as internações terem tendência significativa de queda no tempo tanto neste estado quanto no Mato Grosso do Sul, exceto por infecções respiratórias agudas.

**CONCLUSÕES:**

O COAP continua sendo o instrumento regulador da regionalização vigente, contudo, os resultados obtidos pela adesão a ele na expansão da atenção básica no Ceará e no Mato Grosso do Sul fazem questionar se o modelo contratual, tal como previsto, é o melhor instrumento para o avanço da regionalização do Sistema Único de Saúde.

## INTRODUÇÃO

O Contrato Organizativo de Ação Pública (COAP) foi criado pelo Decreto 7.508/2011^1^ para regulamentar a Lei 8.080/1990 e conferir amparo legal ao processo de coordenação interfederativa do Sistema Único de Saúde (SUS), facilitando, assim, as pactuações necessárias à manutenção das responsabilidades tripartites de gestão da saúde. Entre 2011 e 2016 o Ministério da Saúde empreendeu esforços para construir a pactuação em torno do COAP em todo o país^2^. O modelo contratual, no qual responsabilidades e metas são firmadas entre os entes federativos, foi considerado ideal para acomodar a organização do SUS^3^.

Porém, o COAP foi efetivado apenas para os estados do Ceará e Mato Grosso do Sul. Entre os empecilhos para a finalização do processo de contratualização, destaca-se a dificuldade das secretarias estaduais de saúde em coordenar o processo, a complexidade do instrumento, o financiamento insuficiente, disperso e inadequado para a implantação dos sistemas regionais^2^^,^^4^ e os altos custos de transação na adesão ao contrato^5^. Nos estados onde foi finalizado, entretanto, os impactos sobre a área da saúde não foram estudados até o presente momento. Isso é ainda mais preocupante porque o Decreto 7.508/2011 continua em vigor e, até o momento, é a diretriz de regionalização vigente no SUS.

Este artigo objetiva analisar o impacto da implementação do COAP sobre a ampliação do acesso à atenção básica justamente nos estados onde foi finalizado, Ceará e Mato Grosso do Sul.

## MÉTODOS

### A Intervenção

O COAP é um dispositivo jurídico-executivo introduzido no arcabouço normativo do SUS pelo Decreto 7.508/2011^1^ com o objetivo de regulamentar o planejamento e a articulação interfederativa do sistema de saúde. Somente o Ceará e o Mato Grosso do Sul finalizaram o processo de negociação para a implementação do COAP, que foi negociado com municípios e regiões ao longo de 2012 e entrou em vigor em 2013.

### Desenho e Fontes de Dados

Trata-se de estudo retrospectivo, quantitativo e analítico que utiliza o método de regressão de dados de séries temporais. O estudo terá como fonte dados administrativos do Sistema de Internações Hospitalares do SUS (SIH-SUS) e do Sistema de Cadastro Nacional de Estabelecimentos de Saúde (SCNES), além de dados populacionais provenientes dos censos demográficos do Instituto Brasileiro de Geografia e Estatística (IBGE) realizados ao longo do período estudado e projeções.

### Metodologia de Seleção dos Controles

Optou-se pela construção de modelos utilizando controles recomendados para estudos de séries temporais interrompidas (ITS)^6^, mas, a fim de que os resultados sejam confiáveis, esta abordagem deve utilizar locais estruturalmente semelhantes^7^. Dessa forma, na seleção dos controles utilizou-se um conjunto de indicadores que foram divididos em cinco indicadores socioeconômicos e demográficos, e quatro de sistemas de saúde. O ano de 2011 foi utilizado como referência para obtenção de informações, por ser o último ano antes da implementação do COAP. A única exceção foi o Índice de Desenvolvimento Humano Municipal (IDH-M), calculado com base no Programa das Nações Unidas para o Desenvolvimento (PNUD) 2010.

Os estados brasileiros foram divididos em grupos de acordo com o grau de execução do COAP^4^ e os controles foram escolhidos entre o grupo que teve implementação burocrática, porque foi considerado que estes estados tinham capacidade técnica e política para colocar o programa em prática. A categoria de implementação burocrática é definida pela existência de respostas estaduais concentradas em grupos de momentos encadeados da agenda de implementação. Assim, considera-se que o estado cumpriu a implementação burocrática quando realizou as etapas de Construção de Pacto Inicial, Reconfiguração das Regiões de Saúde e instituiu Comissões Intergestoras Bipartites Regionais (CIR) ou o Planejamento Regional.

Para calcular o índice sumário que norteou a seleção dos controles foi utilizada a diferença entre os valores dos estados de referência – Ceará e Mato Grosso do Sul que tiveram a implementação finalizada – e os valores de outros estados (Fórmula 1). A partir destes valores foi calculada a média da diferença de cada um dos estados e o desvio padrão.

### Fórmula 1

β = *Ec – Er*

Onde: Ec = Estado Controle e Er = Estado Referência

Em seguida, foi calculado, para cada indicador, a quantidade de desvios padrão que cada estado estava da média da diferença, determinando assim a similaridade entre os possíveis controles e os respectivos casos (Fórmula 2).

### Fórmula 2

γ = (β – *M*)/*DP*

Onde: β = Dado da diferença; M = Média; DP = Desvio Padrão

Por fim, o valor absoluto de γ, de cada indicador por estado, foi somado. Os estados que tiveram o resultado da soma mais próximo de zero eram os mais próximos aos estados de referência. Dessa forma, o controle para o Ceará foi o estado de Sergipe (γ = 3,26) e para o Mato Grosso do Sul o controle foi o estado do Mato Grosso (γ = 2,20).

### Seleção dos Indicadores de Desfecho

Foram utilizados para a avaliação de impacto do COAP indicadores classificados como universais no contrato – ou seja, obrigatórios para todos os estados, regiões de saúde e municípios – e cujas informações estivessem disponíveis com acesso livre nos Sistemas de Informação da Saúde (SIS). De modo a avaliar a ampliação do acesso à atenção básica, foram selecionados indicadores de cobertura da atenção básica (CobAB) e a taxa de internações por condições sensíveis à atenção básica (ICSAB), pertencentes à Diretriz 1, Objetivo Nacional 1.1 do COAP^1^, considerando a disponibilidade mensal de dados e sua sensibilidade às ações previstas nos objetivos específicos.

Considerando as taxas de ICSAB, previstas no COAP, calculou-se a taxa de internação segundo os grupos de causas consideradas “agudas”, visando captar aquelas sobre as quais as mudanças ocorridas na atenção básica poderiam ter efeito mais imediato, como gastroenterites, infecções respiratórias agudas (IRA) e asma^8^.

Buscando uma medida mais estável nos indicadores, foram avaliadas periodicidades diferentes. No CobAB utilizou-se a periodicidade mensal, enquanto que, para indicadores de internação, aplicou-se a periodicidade mensal no caso do Ceará e de Sergipe e trimestral para Mato Grosso do Sul e Mato Grosso.

### Análises Estatísticas

Utilizou-se modelos de ITS para analisar o efeito do COAP sobre as variáveis de desfecho. Ao estimar efeitos, estes modelos se ajustam para tendências preexistentes à intervenção. A estimativa Prais-Winsten foi utilizada no *software* STATA v.12 para a execução das análises de regressão.

Os modelos utilizaram dois segmentos temporais, o período anterior à assinatura (janeiro de 2009 a dezembro de 2011) e pós COAP (janeiro de 2013 a dezembro de 2016), considerando 1 ano para a implementação do programa (janeiro a dezembro de 2012). Os efeitos do COAP foram estimados utilizando uma variável representando a mudança de nível das variáveis desfecho imediatamente após sua implementação e outra representando a mudança de tendência pós-intervenção.

Para analisar os efeitos do aumento de CobAB sobre as internações evitáveis ao longo do tempo, foram utilizados modelos de séries temporais não segmentadas, empregando a regressão de Prais-Winsten no STATAv.12.

Em ambos os casos, os parâmetros dos modelos foram preservados independentemente da significância estatística. Resultados com p < 0,05 foram destacados. O ajuste dos modelos para a existência de autocorrelação dos resíduos foi feito utilizando o teste de Durbin-Watson^9^. Também foram testados termos de tendências logarítmicas para acomodar possíveis tendências não lineares durante o segmento pós-intervenção, selecionando o melhor modelo usando os critérios de qualidade critérios de informação Bayesiano (BIC) e de Akaike (AIC)^10^.

Para criar resumos em número único dos efeitos das políticas, a porcentagem de mudança relativa, calculamos as estimativas das mudanças relativas nos resultados de janeiro de 2013, logo após a implementação do COAP, em comparação com os valores esperados com base nas tendências anteriores à implementação do contrato.

Este estudo utiliza apenas bases de dados secundárias de acesso livre e por isso está dispensado de aprovação pelo Comitê de Ética em Pesquisa.

## RESULTADOS

Em 2011 o Ceará tinha um grau de urbanização moderado^11^, Índice de Desenvolvimento Humano (IDH) médio^12^, com índice de envelhecimento e esperança de vida ao nascer mais baixos que a média nacional para o mesmo ano. O controle, Sergipe, compartilhava dessas características, apesar de ter uma população menos envelhecida. Quanto ao sistema de saúde, o número de médicos a cada mil habitantes do Ceará era bem inferior à média brasileira, porém o número de leitos para internação estava bem próximo do nacional. Sergipe apresentava mais médicos por habitante, permanecendo, contudo, abaixo da média nacional, e menos leitos de internação disponíveis. A cobertura de saúde suplementar em ambos os estados era muito similar (abaixo da média nacional). A maior diferença entre os dois está na CobAB, que no Ceará era um pouco inferior àquela do Brasil e em Sergipe era bastante superior.

Mato Grosso do Sul e seu controle, Mato Grosso, tinham alto grau de urbanização, com IDH alto e índice de envelhecimento mais baixo que a média nacional. A esperança de vida ao nascer no Mato Grosso do Sul era exatamente igual à brasileira e a do Mato Grosso estava um pouco abaixo. O número de médicos per capita também era similar entre os estados, ambos abaixo da média nacional, o número de leitos por habitante e a CobAB estavam bem próximos dos valores nacionais para ambos. A cobertura de saúde suplementar era menor que a brasileira em ambos, sendo maior no Mato Grosso do Sul que no Mato Grosso (Tabela 1).

**Tabela 1:** Indicadores sociodemográficos e de rede de atenção à saúde para Brasil (BR), Ceará (CE), Sergipe (SE), Mato Grosso do Sul (MS) e Mato Grosso (MT) em 2011.

		CE	MS	SE	MT	BR
SOCIODEMOGRÁFICOS	Índice de envelhecimento da população	35,3	35,5	29,0	28,8	41,2
	Razão de sexos	95,1	99,3	94,5	104,3	96,0
	Esperança de vida ao nascer	72,7	74,1	71,3	72,9	74,1
	IDH^a^	0,68	0,73	0,67	0,73	0,7
	Grau de urbanização	75,5	85,8	73,6	82,0	84,4
SAÚDE	Número de médicos a cada mil habitantes^2^	0,9	1,4	1,2	1,1	1,9
	Cobertura da atenção básica	66,7	70,1	83,4	67,9	67,1
	Número de leitos para internação a cada mil habitantes^b^	2,2	2,3	1,9	2,2	2,4
	Cobertura da saúde suplementar	12,2	17,2	13,3	12,9	23,5

a Valores com base em PNUD 2010.

b Valores de dezembro de 2011.

Fonte: Censo demográfico e projeções do IBGE, PNUD (2010), Sistema de Cadastro Nacional de Estabelecimentos de Saúde (SCNES) e Sistema de Informação de Beneficiários (SIB/ANS/MS).

Como é possível observar na Tabela 2, a CobAB no período anterior à implementação do COAP (janeiro de 2009 a dezembro de 2011) apresentava leve tendência de queda no Ceará e em Sergipe e também ínfima tendência de aumento no Mato Grosso do Sul e no Mato Grosso. Após a implementação do COAP, Ceará e Mato Grosso do Sul apresentaram aumentos significativos do indicador.

**Tabela 2:** Modelos de análise da efetividade do COAP sobre a CobAB e sobre as taxas de internações evitáveis por meio de ITS.

Indicador	UF	Baseline			Pós COAP (jan. 2013)		Valor em jan. 2013	Mudança relativa (jan. 2013)
		Valor em jan. 2009	Tendência	Valor em dez. 2011	Nível pós COAP (IC95%)	Tendência pós COAP (IC95%)		
	**CE**	78.75	-0.10	75.33	**8.97** **(2.57 a 15.37)**	0.13 (-0.10 a 0.36)	83.16	**4.52**
Cobertura da atenção básica^b^	SE^a^	91.70	-0.09	88.47	1.52 (-2.91 a 5.95)	0.09 (-0.04 a 0.23)	88.89	1.88
	**MS**	65.45	0.01	65.74	**5.26 (1.19 a 9.33)**	0.02 (-0.12 a 0.16)	71.13	**8.63**
	**MT**^a^	68.54	0.06	70.67	**-3.02 (-5.15 a −0.89)**	0.05 (-0.02 a 0.12)	68.50	-6.66
	CE	8.74	-0.03	7.59	0.40 (-1.03 a 1.83)	0.00 (-0.04 a 0.05)	7.57	1.88
Taxa de ICSAB^c^	SE^a^	4.30	-0.02	3.65	0.23 (-0.6 a 1.05)	0.02 (-0.01 a 0.05)	3.65	3.33
	MS	27.43	-0.17	25.54	-0.09 (-4.93 a 4.76)	0.12 (-0.36 a 0.59)	24.72	0.68
	MT^a^	28.77	-0.27	25.78	-1.84 (-4.31 a 0.64)	0.10 (-0.15 a 0.35)	22.69	-1.86
	CE	1.60	-0.02	0.95	0.66 (-0.33 a 1.64)	0.01 (-0.02 a 0.05)	1.37	3.36
Taxa de internação evitável IRA^c^	**SE**^a^	0.34	0.01	0.69	-0.16 (-0.52 a 0.2)	-0.01 (-0.02 a 0)	0.68	**5.79**
	MS	3.99	-0.06	3.31	2.36 (-0.01 a 4.74)	0.03 (-0.22 a 0.28)	5.39	2.07
	**MT**^a^	5.63	-0.31	2.18	**2.45 (0.71 a 4.18)**	**0.31 (0.13 a 0.49)**	3.38	**5.89**
	CE	1.31	-0.01	0.85	0.10 (-0.22 a 0.41)	0.00 (-0.01 a 0.01)	0.78	-3.69
Taxa de internação evitável por asma^c^	SE^a^	0.48	0.00	0.40	0.09 (-0.18 a 0.36)	0.00 (-0.01 a 0.01)	0.46	5.47
	**MS**	3.88	-0.23	1.39	0.78 (-0.72 a 2.29)	**0.19 (0.03 a 0.35)**	1.22	-2.72
	**MT**^a^	3.52	-0.13	2.04	-0.26 (-1.04 a 0.52)	**0.10 (0.02 a 0.18)**	1.21	**2.01**
	CE	2.92	-0.03	1.89	0.69 (-0.55 a 1.93)	0.01 (-0.03 a 0.05)	2.20	4.15
Taxa de internação evitável por gastroenterites^c^	**SE**^a^	1.19	-0.02	0.57	**0.31 (0.03 a 0.6)**	**0.01 (0 a 0.02)**	0.67	**4.29**
	MS	8.45	-0.17	6.59	-0.59 (-3.52 a 2.34)	0.13 (-0.16 a 0.42)	5.29	-0.19
	MT^a^	7.63	-0.04	7.14	-2.23 (-4.97 a 0.51)	-0.04 (-0.31 a 0.23)	4.65	-15.36

ICSAB: internações por condições sensíveis à atenção básica; IRA: internações por infecções respiratórias agudas.

a Casos controle.

b ((N° de ESF + N° de ESF equivalente) x 3.000/população no mesmo local e período) X 100. O cálculo da população coberta pela atenção básica tem como referência três mil pessoas por equipe de atenção básica, conforme a Política Nacional de Atenção Básica (Pnab), (Portaria n° 2.488/11).

c Os modelos de internação para MS e MT foram calculados com dados trimestrais em função da variação mensal.

Fonte: Sistema de Internações Hospitalares (SIH), Sistema de Cadastro Nacional de Estabelecimentos de Saúde (SCNES) e censo demográfico e projeções do IBGE.

Logo após a intervenção, a CobAB no Ceará teve um aumento significativo de 9 pontos percentuais, o que não aconteceu em Sergipe. É possível observar também que o resultado alcançado no Ceará em janeiro de 2013 é significativamente superior (cerca de 4,5%) àquele estimado em caso de continuidade da tendência anterior à implementação do COAP (saindo de 79% para 83%). Em Sergipe, tal qual esperado, não existe uma mudança significativa entre o cenário estimado (contrafacto) e o real.

O nível da CobAB no Mato Grosso do Sul teve um aumento significativo de 5,3 pontos percentuais imediatamente após a intervenção. No Mato Grosso vemos uma queda significativa de 3,0 pontos percentuais entre dezembro de 2011 e janeiro de 2013. O modelo mostra ainda que a cobertura no Mato Grosso do Sul em janeiro de 2013 era 8,6% superior à que seria alcançada em caso de manutenção do cenário pré-implementação (saindo de 65% para 71%).

A efetividade da atenção primária em saúde é frequentemente avaliada a partir de indicadores de internação evitável^13^, e nessa linha o COAP tem previsto um indicador próprio para este fim. No período pré-intervenção era possível observar uma tendência de queda na taxa de ICSAB para todos os estados, que foi alterada para uma tendência de aumento, não significativa, após a intervenção em todos os estados.

A taxa de internação evitável por IRA no período anterior à intervenção tinha uma leve tendência de queda no Ceará e no Mato Grosso do Sul, e discreta tendência de aumento em Sergipe. Mato Grosso apresentava uma tendência de queda mais acentuada no período anterior à 2011. Após a intervenção não encontramos mudanças significativas no Ceará ou em Sergipe, porém, neste vemos um percentual de mudança relativa de 5,8% em relação ao contrafacto. Isso pode ser explicado porque o cálculo da mudança relativa tem como variável o valor do indicador no início da série temporal, e ele dobra no período de 2009 a 2011 (anterior à implementação), o que no cômputo geral é considerado como uma mudança significativa para os valores previstos, porém isso não é associado à intervenção.

Também não foram observadas mudanças significativas após a intervenção no Mato Grosso do Sul. No Mato Grosso vemos aumento de nível e tendência significativos no período após o primeiro trimestre de 2013, com um percentual de mudança relativa de 5,9%.

A taxa de internação evitável por asma apresentava tendência de queda no Ceará, Mato Grosso do Sul e Mato Grosso. Após a intervenção, o modelo mostra tendência de aumento não significativa no Ceará e em Sergipe. No Mato Grosso do Sul vemos uma tendência de aumento significativa no período pós-intervenção, porém esta não se reflete em mudança relativa significativa. No Mato Grosso também é possível observar uma tendência de aumento significativa, com um percentual de mudança relativa de 2,0% (p < 0,05).

Sobre a taxa de internação evitável por gastroenterites, todos os estados apresentavam tendência de queda entre janeiro de 2009 e dezembro de 2011. Após a implementação não vemos mudanças no Ceará, mas em Sergipe o período mostra aumento de nível e tendência significativos, com um percentual de mudança relativa de 4,3%. O modelo não encontrou mudanças significativas para Mato Grosso do Sul e Mato Grosso.

A Figura 1 apresenta a representação visual dos modelos de impacto do COAP sobre a CobAB e a taxa de ICSAB, para casos e controles. É possível observar claramente o aumento de nível da CobAB no Ceará, em detrimento da estabilidade do indicador observada no controle. Da mesma forma, a cobertura no Mato Grosso do Sul aumenta visivelmente de nível, enquanto que no Mato Grosso observamos uma queda acentuada de nível. É importante notar que há relevante diferença entre o comportamento das tendências do caso e do controle pós-intervenção, com estabilidade para o controle e crescimento dos casos.

**Figura 1:** Cobertura da atenção básica, taxa de internação por ICSAB e valores preditos do modelo de regressão segmentada.

Fontes: SIH-SUS e censo demográfico e projeções, IBGE.

Para a taxa de internação por ICSAB, vemos que os cenários pré e pós COAP permanecem praticamente inalterados no Ceará e em Sergipe. É possível ver um pequeno aumento de nível nas internações para o Mato Grosso do Sul e uma diminuição da acentuada tendência de queda presente antes da implementação do COAP, que no modelo estatístico apareceu como tendência de aumento não significativa (Figura 1).

Foram encontrados, portanto, impactos do COAP na CobAB nos casos, mas não uma queda esperada nas taxas de ICSAB. Considerando que a literatura aponta que o aumento na CobAB está correlacionado à diminuição das internações por este conjunto de causas^14^,15, foram elaborados modelos de séries temporais simples para analisar o impacto da expansão da cobertura sobre as internações no tempo.

A Tabela 3 resume os resultados dos modelos de análise da influência da CobAB no tempo sobre as internações evitáveis para o Ceará e Mato Grosso do Sul. Evidenciou-se que, apesar de as internações evitáveis no Ceará declinarem significativamente no tempo em quase todos os conjuntos de causas (com exceção das internações evitáveis por IRA), o aumento da CobAB foi significativo somente na diminuição da taxa de ICSAB.

**Tabela 3:** Modelos de análise da influência da cobertura da atenção básica e tempo sobre as internações evitáveis no Ceará (CE) e no Mato Grosso do Sul (MS), 2009–2016.

Indicador	Cobertura					Tempo			
	UF	Coef.	p > t	IC95%		Coef.	p > t	IC95%	
Taxa de ICSAB	**CE**	**9.27**	**0.04**	**0.51**	**18.03**	**-0.03**	**0.00**	**-0.05**	**-0.02**
	**MS**^a^	0.08	0.75	-0.43	0.59	**-0.19**	**0.05**	**-0.37**	**0.00**
Taxa de internações evitáveis por IRA	CE	1.33	0.55	-3.09	5.75	0.00	0.40	-0.01	0.00
	MS^a^	0.15	0.37	-0.18	0.48	0.03	0.60	-0.08	0.14
Taxa de internações evitáveis por asma	**CE**	0.67	0.49	-0.69	3.07	**-0.01**	**0.00**	**-0.01**	**-0.01**
	**MS**^a^	0.12	0.22	-0.07	0.31	**-0.12**	**0.01**	**-0.16**	**-0.03**
Taxa de internações evitáveis por gastroenterites	**CE**	6.18	0.12	-0.97	15.09	**-0.02**	**0.00**	**-0.04**	**-0.01**
	MS^a^	-0.08	0.63	-0.44	0.27	-0.10	0.11	-0.23	0.02

ICSAB: internações por condições sensíveis à atenção básica; IRA: internações por infecções respiratórias agudas.

a Os modelos de internação para MS foram calculados com dados trimestrais em função da variação mensal.

Fonte: Sistema de Internações Hospitalares (SIH), Sistema de Cadastro Nacional de Estabelecimentos de Saúde (SCNES) e censo demográfico e projeções do IBGE.

No Mato Grosso do Sul os resultados foram similares. Enquanto a taxa de internação cai significativamente no tempo para ICSAB e para asma, o aumento da cobertura não se mostrou significativo em nenhum dos casos (Tabela 3).

Estes resultados podem ser melhor observados na Figura 2, que mostra um salto da CobAB no ano de 2013, especialmente visível no estado do Ceará, e que não é acompanhado de queda igualmente relevante nas taxas de internação. Assim, apesar do aumento de CobAB no estado no período pós-intervenção, a tendência de queda na taxa de ICSAB desacelera no mesmo período, só voltando a cair mais acentuadamente a partir de março de 2015. A taxa de internação evitável por asma no Ceará não parece ter sido alterada. No Mato Grosso do Sul também é possível observar aumento de nível na CobAB que não é acompanhado por nenhuma alteração na tendência de queda da taxa de ICSAB ou de asma.

**Figura 2:** Cobertura da atenção básica e resultados dos modelos de regressão para internações evitáveis (total e asma) no Ceará (CE) e no Mato Grosso do Sul (MS), 2009–2016.

Fonte: SIH-SUS, SCNES-SUS e censo demográfico e projeções

## DISCUSSÃO

Este artigo utiliza ITS para verificação do impacto do COAP no Ceará e no Mato Grosso do Sul. A análise utilizou casos controle para os dois estados, pois a utilização de controles é importante quando empregamos o ITS em razão da dificuldade de inferir causalidade entre os padrões de dados encontrados e a intervenção estudada^6^.

Os resultados mostraram que a implementação do COAP teve impacto positivo sobre o aumento da cobertura nos dois casos, o que não aconteceu nos controles. Contudo, esse impacto não se refletiu na diminuição das taxas de ICSAB ou de internações por causas evitáveis agudas. Quando analisamos os efeitos do aumento de CobAB sobre as internações evitáveis entre 2009 e 2016, observamos que a cobertura teve impacto positivo na diminuição da taxa de ICSAB apenas no Ceará, apesar de as internações apresentarem tendência significativa de queda no tempo tanto no Ceará quanto no Mato Grosso do Sul, exceto por IRA.

Os modelos mostram que imediatamente após a implementação do COAP, o nível de CobAB aumenta significativamente no Ceará e no Mato Grosso do Sul. Uma vez que o aumento ocorre em janeiro de 2013 e não ocorre nos controles, podemos descartar que esses resultados sejam uma confusão com possíveis impactos de programas concorrentes, principalmente o Programa Mais Médicos, iniciado em julho de 2013^16^.

A cobertura é o indicador mais sensível para avaliar o aumento do acesso aos serviços básicos de saúde, uma vez que mede justamente a disponibilidade desses serviços no território. Esse dado é importante, pois indica uma ampliação do acesso aos serviços de saúde, uma vez que a atenção básica é, no Brasil, a principal estratégia para coordenar os cuidados na rede^17^.

A taxa de internação por ICSAB, por sua vez, é bastante utilizada para medir o desempenho da atenção básica, considerando que altas taxas de hospitalização por determinadas doenças refletem problemas e dificuldades de acesso a serviços de saúde e baixa resolutividade da atenção básica^13^.

Em razão do tipo de intervenção, seria esperado que não houvesse mudança de nível das internações por ICSAB logo após a implementação, uma vez que o aumento da CobAB e a diminuição das taxas não ocorrem simultaneamente^18^. O que poderíamos esperar era um aumento da tendência de queda após a implementação do COAP, porém isso não foi observado.

Os modelos não mostraram impacto significativo do COAP sobre a diminuição das taxas de ICSAB ou sobre as taxas de internação por causas evitáveis agudas. Todavia, considerando os resultados dos modelos que apontam que o COAP teve impacto sobre o aumento da CobAB nos casos, foram elaboradas análises para estimar os efeitos específicos do aumento de CobAB sobre as internações evitáveis entre 2009 e 2016.

A taxa de ICSAB no Ceará cai significativamente tanto no tempo quanto em relação ao aumento da CobAB. Isso corrobora outros estudos sobre o estado, que correlacionaram o aumento da cobertura da Estratégia de Saúde da Família à queda da taxa de Internações por Condições Sensíveis à Atenção Primária (ICSAP)^19^. A taxa de ICSAB no Mato Grosso do Sul cai significativamente no tempo, mas não está correlacionada à expansão da CobAB. Isso indica que existem outros elementos determinantes da hospitalização, que estão fora do escopo de atuação da atenção básica à saúde, tais como, características inerentes ao paciente, fatores socioeconômicos e demográficos, variabilidade da prática clínica hospitalar, e políticas de admissão nesses serviços^20^,21, além do modelo de organização da atenção em saúde^19^,22. Neste sentido, a taxa de ICSAB é um indicador menos sensível e específico ao fenômeno analisado^23^.

As taxas de internação evitáveis por IRA não se mostraram associadas significativamente ao tempo e ao aumento da CobAB em ambos os estados. Por outro lado, as internações por asma caem significativamente no tempo nos dois casos. Isso pode estar relacionado à distribuição de medicamentos para asma de forma gratuita pelo Programa Farmácia Popular a partir de junho de 2012^24^.

Destaca-se como novidade do presente estudo a aplicação do método ITS, importante ferramenta na mensuração do efeito de políticas públicas^25^,26. A maior parte dos artigos sobre o tema é direcionada a compreender os motivos para a baixa adesão ao COAP^2^,4,5,27. Contudo, na percepção dos gestores estaduais do Ceará, os resultados alcançados ficaram aquém dos esperados^28^.

Sergipe foi escolhido como controle devido a sua maior similaridade matemática, considerando os indicadores selecionados. Porém, como limitação, destaca-se a possibilidade de haver um “efeito teto” para o indicador de CobAB e possível “efeito chão” nas internações por IRA e asma.

Ressalta-se lacuna na literatura relativa à análise das ICSAB ou ICSAP para o Mato Grosso do Sul no período de referência do presente estudo, a despeito da discussão ser encontrada para todos os outros estados do país.

Este artigo avaliou apenas a expansão da atenção básica frente ao COAP. Por isso, sugere-se a realização de estudos similares para avaliar o impacto do Contrato sobre demais dimensões. O COAP não é a primeira tentativa de criar um instrumento para mediar a regionalização em saúde (por exemplo, NOB-SUS 01/91, 01/93 e 01/96; NOAS-SUS 01/01 e 01/02; Pacto pela Saúde), assim a utilização do ITS pode ser uma ferramenta para avaliar as tentativas anteriores de integração regional do SUS.

## CONSIDERAÇÕES FINAIS

A bem sucedida descentralização do SUS ao longo da década de 1990 transferiu para os municípios grande parte das funções de gestão de recursos e de implementação de políticas e programas de saúde^4^. O aprofundamento deste processo levou à fragmentação do sistema de saúde e à necessidade de criação de instrumentos que garantissem a organização de redes de atenção^5^.

O COAP foi adotado em 2011, após as diversas tentativas desenvolvidas nesse sentido durante as décadas de 1990 e 2000 não alcançarem os resultados esperados^3^,27, pois, em tese, seria uma proposta mais compatível com o modelo federativo, uma vez que permitiria que os entes federados na região de saúde se autorregulassem, definindo, eles mesmos, a repartição de competências executiva, orçamentário-financeira e de controle e avaliação.

A adesão ao COAP foi, contudo, muito baixa. O Ministério da Saúde não foi capaz de criar uma cadeia de incentivos que tivesse poder de convencimento e indução junto aos demais envolvidos na contratualização^2^,5, assim como de se organizar internamente para reger com coesão o processo de regionalização^28^. Apesar das iniciativas de criação das redes temáticas de saúde, como o Projeto QualiSUS-Rede, estas não tiveram relação com o COAP ^29^.

Este estudo mostrou que o COAP teve impacto positivo sobre a CobAB, um indicador de estrutura, naturalmente mais sensível à atuação direta do gestor público. Isso não se traduziu sobre a diminuição das taxas de ICSAB, indicador de desempenho designado no próprio COAP, que é, no entanto, menos sensível e específico ao tipo de mudança que está sendo analisada.

Em dezembro de 2016 tanto Ceará quanto Mato Grosso do Sul não renovaram o COAP, o que exauriu a iniciativa. Entretanto, este continua sendo o instrumento regulador da regionalização vigente. Considerando que a necessidade de coordenação regional continua existindo, e que alguns projetos de lei buscam reativá-lo^30^^,^^31^, a adequação do modelo contratual para o avanço da regionalização do SUS deve ser avaliada na perspectiva do custo-benefício, avaliando, por um lado, a baixa adesão ao COAP provocada pelos altos custos de transação e, por outro, os resultados obtidos pela adesão sobre a expansão da atenção básica no Ceará e no Mato Grosso do Sul.
